# Disorders of spermatogenesis: Perspectives for novel genetic diagnostics after 20 years of unchanged routine

**DOI:** 10.1007/s11825-018-0181-7

**Published:** 2018-02-26

**Authors:** Frank Tüttelmann, Christian Ruckert, Albrecht Röpke

**Affiliations:** Institute of Human Genetics, University of Münster, Vesaliusweg 12–14, 48149 Münster, Germany

**Keywords:** Male infertility, Oligozoospermia, Azoospermia, Männliche Infertilität, Oligozoospermie, Azoospermie

## Abstract

Infertility is a common condition estimated to affect 10–15% of couples. The clinical causes are attributed in equal parts to the male and female partners. Diagnosing male infertility mostly relies on semen (and hormone) analysis, which results in classification into the two major phenotypes of oligo- and azoospermia. The clinical routine analyses have not changed over the last 20 years and comprise screening for chromosomal aberrations and Y‑chromosomal azoospermia factor deletions. These tests establish a causal genetic diagnosis in about 4% of unselected men in infertile couples and 20% of azoospermic men. Gene sequencing is currently only performed in very rare cases of hypogonadotropic hypogonadism and the *CFTR* gene is routinely analysed in men with obstructive azoospermia. Still, a large number of genes have been proposed to be associated with male infertility by, for example, knock-out mouse models. In particular, those that are exclusively expressed in the testes are potential candidates for further analyses. However, the genome-wide analyses (a few array-CGH, six GWAS, and some small exome sequencing studies) performed so far have not lead to improved clinical diagnostic testing. In 2017, we started to routinely analyse the three validated male infertility genes: *NR5A1, DMRT1*, and *TEX11*. Preliminary analyses demonstrated highly likely pathogenic mutations in these genes as a cause of azoospermia in 4 men, equalling 5% of the 80 patients analysed so far, and increasing the diagnostic yield in this group to 25%. Over the past few years, we have observed a steep increase in publications on novel candidate genes for male infertility, especially in men with azoospermia. In addition, concerted efforts to achieve progress in elucidating genetic causes of male infertility and to introduce novel testing strategies into clinical routine have been made recently. Thus, we are confident that major breakthroughs concerning the genetics of male infertility will be achieved in the near future and will translate into clinical routine to improve patient/couple care.

## Zusammenfassung

Etwa 10–15 % aller Paare erzielen auf natürlichem Weg keine Schwangerschaft und sind nach WHO-Definition als „sub-/infertil“ einzustufen. Klinische Ursachen werden bei diesen Paaren etwa zur Hälfte bei der Frau bzw. beim Mann nachgewiesen. Die klinischen Untersuchungen bei männlicher Infertilität beschränken sich derzeit auf Ejakulat- und Hormonuntersuchungen, die dann bei einer Vielzahl der Männer zur deskriptiven „Diagnose“ Oligozoo- oder Azoospermie führen, wodurch die Ursache der Infertilität des Paares erklärt werden kann. Der eigentliche Grund für die Spermatogenesestörung bleibt damit aber unklar. Die genetische Diagnostik bei infertilen Männern hat sich in den letzten 20 Jahren nicht weiter entwickelt und umfasst nach wie vor ausschließlich das Screening hinsichtlich Chromosomenstörungen und Y‑chromosomaler AZF-Deletionen. Diese beiden Untersuchungen finden die tatsächliche Ursache der Spermatogenesestörung bei etwa 4 % der unselektierten Männer und bei etwa 20 % der Männer mit Azoospermie. Gensequenzierungen werden hingegen bislang ausschließlich bei Patienten mit hypogonadotropem Hypogonadismus, einem umschriebenen, sehr seltenen Krankheitsbild, bzw. bei obstruktiver Azoospermie (*CFTR*-Analytik) durchgeführt. Andererseits wurden bereits viele Gene publiziert, in denen Mutationen potenziell zu einer Infertilität des Mannes führen können. Allerdings haben die bislang publizierten Daten und auch die genomweiten Analysen keine Erweiterung der klinischen Diagnostik erreicht. Seit Anfang 2017 haben wir drei Kandidatengene – *NR5A1*, *DMRT1* und *TEX11* – bei Männern mit Azoospermie sequenziert. Die vorläufigen Auswertungen ergaben vier wahrscheinlich pathogene Mutationen in diesen Genen. Dies entspricht 5 % der 80 bislang ausgewerteten Männer. Die kausalen Diagnosen steigen bei dieser Patientengruppe somit auf etwa 25 %. In den vergangenen Jahren wurden zunehmend weitere Kandidatengene publiziert. Gleichzeitig laufen mehrere große Studien bei infertilen Männern. Deswegen gehen wir davon aus, dass in naher Zukunft weitere klinisch relevante Erkenntnisse gewonnen werden, die dann auch Einzug in die Routinediagnostik finden und die Behandlung dieser Männer bzw. des Paares verbessern werden.

## Introduction

Infertility, which has been defined by the WHO as inability to conceive after 1 year of unprotected intercourse, is a common condition estimated to affect 10–15% of couples in developed and developing countries [56]. The clinical causes are attributed in equal parts to the male and female partners, with about 30% of couples having reduced fertility potential in both partners. In otherwise healthy men, infertility is primarily diagnosed by semen analysis comprising determination of sperm concentration/total count, motility and morphology. Lower reference ranges for these and other semen parameters have been determined in recent years from a “normal” population of men that induced a pregnancy within 1 year (Table 1) and have been published by the WHO [55].

**Table 1:** The most important WHO reference ranges for semen analysis

Semen parameter	Reference range
Semen volume	≥1.5 ml
pH	≥7.2
Sperm concentration	≥15 million sperm/ml
Total sperm count	≥39 million sperm/ejaculate
Total sperm motility	≥40% motile sperm
Progressive sperm motility	≥32% progressively motile sperm (former categories a + b)
Sperm morphology	≥4% morphologically normal sperm

**Fig. 1:**
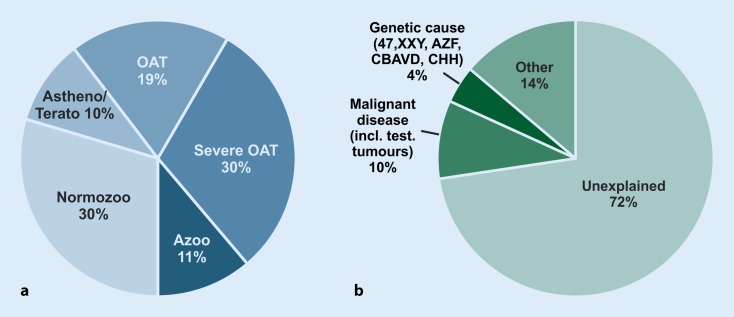
**a** Descriptive diagnoses according to semen analyses of 26,091 men in infertile couples who attended the Centre of Reproductive Medicine and Andrology (CeRA), Münster over the last 30 years. **b** Clinical diagnoses in the same men. Data from Androbase^©^, the clinical patient database [46]

In most cases, male infertility is clinically diagnosed if semen parameters are reduced. Descriptive diagnoses are “oligozoospermia” (reduced sperm count), “asthenozoospermia” (reduced sperm motility), “teratozoospermia” (reduced percentage of sperm with normal morphology). Combinations are common; most frequently “oligoasthenoteratozoospermia” or “OAT syndrome” are found. The most severe clinical phenotype is “azoospermia”, i. e. no sperm are found in the ejaculate even after centrifugation. The frequency of these phenotypes varies significantly between primary care practice and specialised centres. For example, a tertiary care centre such as the Centre of Reproductive Medicine and Andrology (CeRA), Münster, is consulted by a significantly higher number of azoospermic men, because testicular biopsies to obtain spermatozoa are performed there. The distribution of phenotypes from semen analyses of men in infertile couples attending the CeRA is shown in Fig. 1a.

**Fig. 2:**
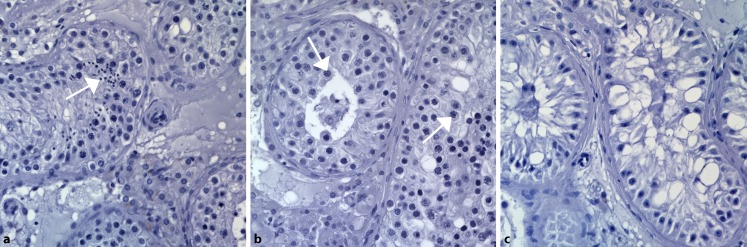
Histological images (CeRA) of human testicular tissue sections from patients with (**a**) obstructive azoospermia and quantitatively and qualitatively normal spermatogenesis, **b** meiotic arrest, and **c** Sertoli cell-only syndrome. Most advanced germ cell types (**a** elongated spermatids, **b** spermatocytes) are indicated by *white arrows*

Semen analysis should be accompanied by measurement of serum hormone levels of at least the pituitary-produced gonadotrophins luteinising hormone (LH) and follicle-stimulating hormone (FSH) in addition to testosterone [48]. If spermatogenesis is reduced, FSH increases because of the hypothalamic–pituitary–gonadal feedback loop. Thus, in a large fraction of about 60% of infertile men, hypergonadotropic oligo- or azoospermia are found. Men with this type of severe spermatogenic failure may also exhibit reduced testicular volume, decreased serum testosterone and increased LH levels as a sign of broader testicular dysfunction, i. e. hypogonadism. Hypergonadotropic azoospermia can also be termed “non-obstructive azoospermia” (NOA). In contrast, obstructive azoospermia (OA) is suspected if FSH levels and testicular volume are normal. OA is mainly caused by the physical blockage of the male excurrent ductal system. Affected men have quantitatively and qualitatively normal spermatogenesis (Fig. 2a). Obstructive azoospermia is most commonly caused by mutations in the *CFTR* gene, which lead to incomplete formation of the vas deferens and congenital bilateral absence of the vas deferens (CBAVD), an association first described 50 years ago [19].

Azoospermia, which can be considered the clinically most severe phenotype of male infertility because natural conception cannot occur, has been estimated to affect 0.1 to 1% of all men and 10–15% of men in infertile couples [50]. In men with azoospermia, the definitive (albeit still descriptive) diagnoses can only be determined by testicular biopsy, which is usually performed to obtain spermatozoa (testicular sperm extraction, TESE). These are needed for intracytoplasmic sperm injection (ICSI), one form of assisted reproductive technology (ART), where one sperm is injected into an oocyte. The most common histological classifications are:

1. “Mixed atrophy” (tubules with varying stages of spermatogenesis).

2. Various types of “spermatogenic arrest” (such as round spermatid or meiotic arrest, MA, Fig. 2b, these stages being the most advanced that can be found).

3. “Sertoli cell-only syndrome” (SCOS, in which the tubules contain no germ cells at all, Fig. 2c).

These can be global (present in all tubules) or focal, with a variable percentage of tubules displaying various stages of qualitatively and quantitatively limited spermatogenesis [5].

All of the descriptive categories mentioned help to classify the “male factor” in couple infertility, but do not offer any causal diagnoses for disturbed spermatogenesis (or causes of obstruction) in the affected men. However, elucidating the cellular/molecular cause of spermatogenic impairment is rather difficult. The testis is not only composed of the two distinct compartments of the interstitium (containing amongst others the testosterone-producing Leydig cells) and the seminiferous tubules (containing the somatic Sertoli cells and the germ cells), but spermatogenesis is one of the most complex differentiation processes in which cells transform from spermatogonia through several stages to mature spermatozoa and undergo meiosis in between. Accordingly, spermatogenesis is thought to be orchestrated by a multitude of up to 2000 genes, of which 600 to 900 seem to be exclusively expressed in the male germline [7, 28, 39, 57]. Thus, the genetics of “male infertility” are difficult to tackle.

## Current clinical diagnoses and genetic routine analyses

Male infertility can be caused by genetic defects that increase in prevalence when spermatogenesis is severely impaired. Currently, a specific causal diagnosis can be attributed to about 28% of unselected infertile men according to our own large dataset. Only very few comparable, large-scale epidemiological studies are available that address this topic, but they report frequencies in the same order of magnitude [32]. These mostly consist of previous gonadotoxic chemo- or radiotherapy for the treatment of malignant disease (including testicular tumours; ~10%) and several other causes such as general/chronic diseases (e. g. diabetes) or testosterone abuse (~14%). Currently, only about 4% of causal genetic diagnoses can be established (Fig. 1b). These comprise structural and numerical chromosomal aberrations (e. g. Klinefelter syndrome; karyotype 47, XXY), microdeletions of the aoospermia factor (AZF) regions on the long arm of the Y chromosome, and mutations of the *CFTR* gene in obstructive azoospermia (for further reading see Tournaye et al. [45]). AZF microdeletions have been reported in highly variable prevalence depending on geographic origin and on selection criteria. It has been shown several times that the deletion frequency in Germany seems to be rather low in comparison to other regions [22, 40]. In addition, a large number of genes involved in the migration and function of GnRH neurons or their hypothalamic targets have been discovered that may be mutated in patients with congenital hypogonadotropic hypogonadism (CHH) with or without anosmia (for current reviews see [6, 38]). In azoospermic men, the genetic diagnostic yield increases to about 20% (Table 2). All of these causes can be identified by well-established genetic tests and form the widely applied clinical routine analyses. Other genetic causes of male infertility comprise disorders of androgen action, genetic syndromes that include infertility as a symptom, and specific defects of sperm morphology and function. Furthermore, mutations and polymorphisms of various genes have been found to be associated with unspecific spermatogenic failure/male infertility, but none of these has been introduced into the clinical work-up of infertile males so far. Thus, in about 72% of men in infertile couples, no causal diagnoses can be established and the aetiology of disturbed spermatogenesis remains largely unclear.

**Table 2:** Genetic causes identified by current routine analyses (patients of the Centre of Reproductive Medicine and Andrology [CeRA] Münster)

Genetic diagnosis	Unselected patients (*N* = 26,091) (%)	Azoospermic patients (*N* = 3252) (%)
*Chromosomal aberrations*	*2.8*	*15.0*
Klinefelter syndrome (47, XXY)	2.6	13.7
XX-Male (46, XX)	0.1	0.6
Translocations	0.1	0.3
Others	<0.1	0.4
*Isolated congenital bilateral absence of the vas deferens or cystic fibrosis*	*0.5*	*3.1*
*Congenital hypogonadotropic hypogonadism* *including Kallmann syndrome*	*0.7*	*0.9*
*Y-chromosomal azoospermia factor deletions*	*0.3*	*1.6*
**Total**	**4.3**	**20.6**

## Monogenic causes of spermatogenic failure

In the field of male infertility, sequencing of genes in clinical setting is currently performed in the very rare condition of CHH, in which gene panels have been introduced into clinical routine in the last few years [6, 41]. In addition, analysis of the *CFTR* gene is routinely performed in men with obstructive azoospermia and mutations in the *ADGRG2* gene have been recently described to cause a similar phenotype [8]. However, for most patients with the common phenotypes of oligo- and azoospermia, no specific genetic sequencing strategy exists thus far. Indeed, no genetic causes relevant to the clinical diagnostic work-up, treatment decisions or counselling with regard to the reproductive health of offspring have been identified in over 20 years [11, 29, 30, 48] when AZF deletions were described as a common cause of spermatogenic failure [54]. This is especially surprising because it was estimated long ago that overall about 30% of cases of male infertility are caused by chromosomal abnormalities or mutations of genes involved in germ cell production and function [53] and familial clustering of male infertility was shown in some case–control studies [16, 17, 25]. In the case of azoospermia in particular, a genetic origin can be suspected in most affected men. Thus, there is a large gap of genetic diagnoses ranging from ~25% in unselected infertile men to ~70% in azoospermic men. This may be partially explained by two important differences in comparison to studying other phenotypes:

(1) Classical linkage analysis or association studies are difficult in infertility because large families with infertility are by nature uncommon.

(2) The parents of an infertile man (and woman) – as the rest of the family – are usually not informed of a patient’s problem conceiving a child.

**Fig. 3:**
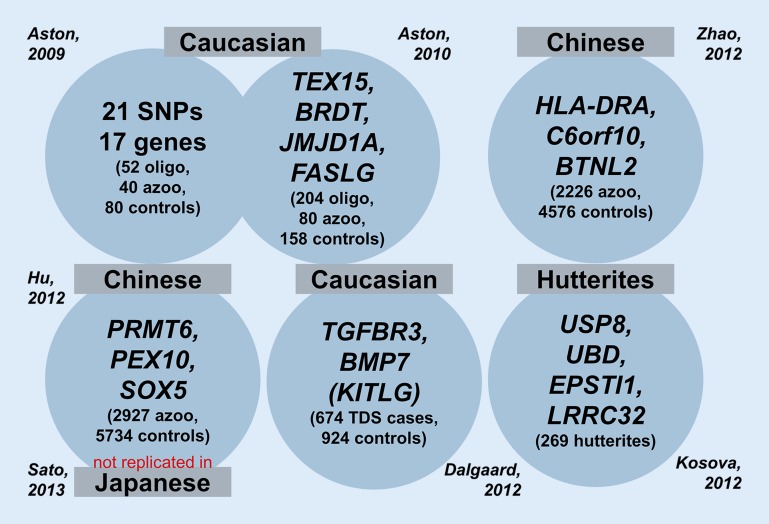
Genome-wide association studies (GWAS) in male infertility show no overlap between identified candidate genes so far (number of cases/controls analysed in brackets). *oligo* oligozoospermia, *azoo* azoospermia, *TDS* testicular dysgenesis syndrome

As described above, male infertility should be considered a complex, multifactorial and clinically and genetically heterogeneous disease. Not surprisingly, single candidate gene approaches did not identify novel genetic causes of infertility [4, 21]. At the level of single-nucleotide polymorphisms (SNPs), our review and meta-analysis from 2007 did not provide any clinically significant associations [47]. Likewise, during the last few years, six genome-wide association studies (GWAS) of highly variable numbers of patients did not reveal an overlap among the highest ranking genes that were reported to be “associated with male infertility” (Fig. 3; [1, 2, 10, 18, 20, 38, 60]). Moreover, either no replication studies have been performed so far or mostly did not confirm the identified candidate genes. Reasons probably include:

1. Genetic variants negatively affecting male reproductive fitness are selected against during evolution and are, therefore, not included in the set of common SNPs used in GWAS.
2. The cohorts were too small to detect genetic variants with a small effect size, and/or
3. Patient selection was too broad owing to poorly defined phenotypes (“men with azoospermia”, testicular histology not known).

Overall, genome-wide approaches with the aim of identifying novel candidate genes have not been applied frequently in male infertility. Still, and as in other genetic disorders, the power of such approaches has been demonstrated by genome-wide array-comparative genomic hybridisation (array-CGH) in groups of clinically well-characterised oligo- and azoospermic men. We were the first to report an excess of copy number variations (CNVs) especially on the sex-chromosomes [49], which has been confirmed by others [15, 27]. However, aside from *DMRT1* (see above) and *TEX11* (see below) no deletions in genes have yet been confirmed in independent studies.

**Fig. 4:**
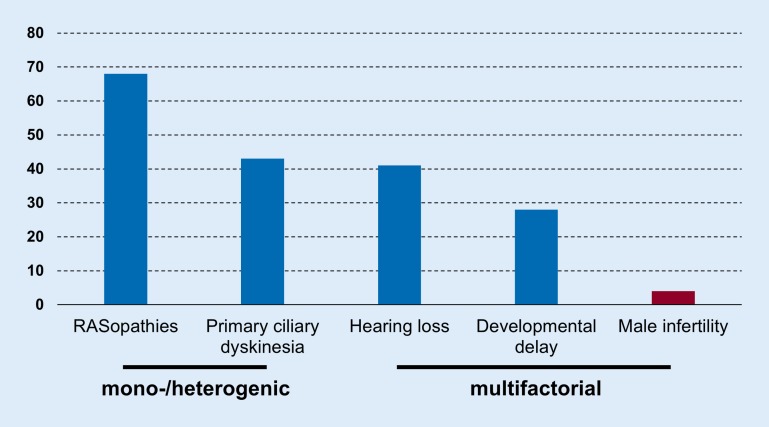
Detection rates of molecular diagnostic tests (%; adapted from Rehm [35])

In other heterogeneous diseases, such as RASopathies and primary ciliary dyskinesia (PCD), and multifactorial diseases, such as hearing loss, large advances in genetic diagnostics have been observed in the last few years because of the recent technological developments of large scale sequencing approaches made available through next-generation sequencing (NGS). Consequently, the genetic diagnostic yield has increased to about 30% even in polygenic multifactorial diseases such as intellectual disability, which is in stark contrast to male infertility (Fig. 4). Taking this into account, large-scale whole-exome sequencing (WES) studies are currently lacking in male infertility and only very few novel candidate genes have been described, mostly in small studies, sometimes in single consanguineous families. Current examples are *TEX15* [31] and *NPAS2* [33] in non-obstructive azoospermia and *ADGRG2* [8] in obstructive azoospermia.

## Towards a gene panel for male infertility

To date and to our knowledge, only three genes have been identified that fulfil the following criteria:

1. Biological evidence for the putative association with male infertility (e. g. knock-out mouse model shows male infertility).
2. Replicated in an independent study.
3. Functional evidence that identified variants are pathogenic.

### NR5A1

The gene *NR5A1* (nuclear receptor subfamily 5, group A, member 1, OMIM 184757) encodes the steroidogenic factor 1 (SF1) protein. Mutations in *NR5A1* are well known to cause autosomal-dominant primary adrenal insufficiency and 46, XY disorders of sexual development, and later also in men with hypospadias, bilateral anorchia and micropenis in addition to women with primary ovarian insufficiency [13]. In 2010, heterozygous missense mutations were found in 4% of French infertile men with unexplained reduced sperm counts, but all mutation carriers were of non-Caucasian ancestry [3]. Therefore, we performed a comprehensive *NR5A1* sequence analysis in almost 500 well-characterised and predominantly Caucasian patients with azoospermia or severe oligozoospermia [37]. Along with several synonymous variants of unclear pathogenicity, three rare heterozygous missense mutations were identified that were affecting conserved amino acids and predicted to be damaging to SF1 protein function. The semen phenotype of mutation carriers seems variable, but all three men had azoospermia or severe oligozoospermia (sperm concentration below 1 million/ml). Overall, the mutation frequency in our patient group was about 1%, depending on the subgroups analysed. Another study in Italian men confirmed *NR5A1* mutations as a cause of severe spermatogenic failure [12].

Of note, clearly detrimental *NR5A1* (nonsense) mutations or deletions are not expected in this group of infertile but otherwise healthy men because such mutations would cause the more severe phenotypes mentioned above. Functional evidence that missense mutations actually impair SF1 transcriptional activity on target genes compared with wildtype SF1 has been provided in at least two independent studies [3, 12].

### DMRT1

The gene *DMRT1* (doublesex- and MAB3-related transcription factor 1, OMIM 602424) encodes another transcription factor that plays a key role in testis differentiation and is expressed mainly in the testes. Deletions of the short arm of chromosome 9 encompassing *DMRT1* are well-known to be associated with 9p deletion syndrome and XY gonadal dysgenesis [23, 52]. Consecutively, in 2013, smaller deletions in *DMRT1* were identified in five infertile men with azoospermia but no symptoms of gonadal dysgenesis [27]. At the same time, we hypothesised *DMRT1* to be an interesting candidate gene for male spermatogenic failure and sequenced this gene in around 300 infertile patients with azoo- or cryptozoospermia (sperm concentration below 0.1 mill/ml) [43]. In total, we detected four rare, putative pathogenic missense mutations in six patients (3.5%), two of which, however, were also found in controls (men with normal spermatogenesis). Those two mutations not detected in controls were exclusively found in men with azoospermia (~1%). Another study screened azoospermic men for *DMRT1* exonic insertions and deletions (by MLPA, *n* = 68) and point mutations (by Sanger sequencing, *n* = 155) and found only non-coding or synonymous substitutions. However, these were overrepresented in patients when compared with almost 400 controls [26]. To date, it remains to be clearly demonstrated whether heterozygous mutations or deletions in *DMRT1* are sufficient to cause gonadal dysgenesis or spermatogenic failure. Although the same problem of mostly identifying missense mutations, which are more difficult to interpret per se (see above), *DMRT1* remains one of the highest ranking candidate genes for both conditions.

### TEX11

In a collaborative study involving our colleagues from the Magee-Womens Research Institute, Pittsburgh, PA, USA (led by Alexander Yatsenko), the CeRA and ourselves, mutations in the X‑linked gene *TEX11* (testis-expressed gene 11, OMIM 300311) were identified to be a cause of meiotic arrest and azoospermia [59]. In the first step of this study, high-resolution array-CGH was used to screen men with non-obstructive azoospermia, revealing a recurring deletion of three exons of *TEX11* in two patients. Because TEX11 protein was previously shown to be required for completing meiosis in a knock-out mouse model, it immediately became an interesting candidate for further analysis. By sequencing *TEX11* in a larger group of almost 300 azoospermic men, more clearly pathogenic (truncating and splice) mutations were detected, whereas no mutations were found in controls. Overall, mutations in *TEX11* were identified in more than 2% of azoospermic men and in as many as 15% of patients with meiotic arrest. This breakthrough relied on the combination of genetics and phenotyping by testicular histology. *TEX11* mutations were already confirmed to be a common cause of azoospermia in an independent study by Yang et al. [58]. The authors also provided evidence that TEX11 protein levels modulate genome-wide recombination rates in both sexes. Thus, hemizygous mutations in the *TEX11* are to date the first X‑chromosomal and major single gene defect in azoospermia.

## First results of a small gene panel of *NR5A1, DMRT1*, and *TEX11*

In preparation for the recently established Clinical Research Unit “Male Germ Cells: from Genes to Function” (German Research Foundation, DFG CRU326), we expanded our analyses of clinically well-characterized men with unexplained azoospermia who attended the CeRA. Patients with known causes of male infertility, such as chemo- or radiotherapy, were excluded in advance. Since January 2017, a total of 323 men with unexplained azoospermia presented at the CeRA for the first time. Initially, the routine chromosomal and AZF analyses were performed. Overall, 46 patients (~14%) were identified with numerical (almost exclusively 47, XXY) or structural aberrations (46, XX; aberrant Y chromosomes; translocations; inversions). AZF deletions were found in almost 2% (6 out of 310).

In a second step, sequence analysis of three genes, *NR5A1, DMRT1*, and *TEX11*, was offered and carried out in consenting men with apparently normal karyotypes and without AZF deletions. Up to December 2017, over 150 men agreed to participate about 80 of whom have been analysed thus far. All non-polymorphic variants (i. e. rare, below 1% minor allele frequency in public and our in-house databases) were strictly classified under clinical conditions according to the American College of Medical Genetics and Genomics guidelines for the interpretation of sequence variants [36]. Potentially pathogenic variants were identified in the *DMRT1* and *TEX11* genes (one each) and two different mutations in the *NR5A1* gene (in one man each).

In conclusion, the basic genetic analyses in men with non-obstructive azoospermia using conventional cytogenetic analysis and AZF screening revealed the expected number of aberrations. Sequencing of these three genes, which have been confirmed to be responsible for spermatogenetic failure, a highly likely cause of azoospermia, could be demonstrated in 4 patients, equalling 5% of the patients analysed so far and increasing the diagnostic yield in this patient group to 25%.

## Outlook: clinical relevance, recent progress, concerted actions

To date, genes that have been found to be mutated in infertile men in one study have mostly not been validated in an independent study. Several potential drawbacks may serve as explanation: patient selection was too broad because of poorly defined phenotypes (e. g. “men with azoospermia”, but testicular histology was not known) and/or the number of patients analysed was too small to detect rare genetic variants. The latter seems especially important as it becomes increasingly clear that aside from X‑chromosomal causes (e. g. *TEX11*) [59] and consanguineous families with probably very rare autosomal-recessive causes (e. g. *TEX15*) [31], autosomal-dominant causes may constitute the majority in male infertility. Thus, rare de novo mutations with dominant effects may well explain a large fraction of non-obstructive azoospermia and potentially also milder forms of male infertility such as oligozoospermia. This would be comparable with many other common, genetically highly heterogeneous diseases such as intellectual disability [24, 34, 51].

All of the currently established genetic diagnoses in infertile males have direct prognostic value for the patients and for the health of the offspring [44, 45]. Men with CBAVD carrying *CFTR* mutations have very high chances of successful testicular sperm extraction (TESE), but also significantly increased risks for cystic fibrosis (CF) in their children, depending on the *CFTR* carrier status of their partner. Men with Klinefelter syndrome, previously thought to have no chance of fathering children, now have an estimated chance of around 50% of successfully obtaining spermatozoa by (microsurgical) TESE. Depending on the type of deletion, azoospermic men carrying AZF deletions have virtually zero (AZFa/b) to up to 50% (AZFc) chance of TESE and their sons will inherit the deletion and likely also be infertile [22]. Thus, even though the detection of a genetic alteration does not substantially change the treatment in most cases, the clinical value lies in:

1. Establishing a definitive causal diagnosis.

2. The prognostic value comprising chances of testicular sperm extraction and pregnancy.

3. Assessing the risks for the offspring in the case of successful treatment.

During the last few years, we observed a steep increase in publications on novel candidate genes for male infertility, especially in men with azoospermia. This is mostly due to application of NGS strategies, and, in comparison with earlier times, better characterised patient groups. Examples comprise the already mentioned *TEX15, NPAS2*, and *ADGRG2* genes. However, regarding all other previously proposed candidate genes, such as *SOHLH1* [9], *USP26* [42], *MEIOB, TEX14*, and *DNAH6* [14], validation in another (ideally larger) study is urgently warranted.

Fortunately, concerted efforts to achieve progress in elucidating genetic causes of male infertility and introduce novel testing strategies into clinical routine have been recently established. Don Conrad (Washington University School of Medicine, St. Louis, MI, USA) and Ki Aston (University of Utah, Salt Lake City, UT, USA) lead the NIH-funded “Genetics of Male Infertility Initiative” (GEMINI, http://gemini.wustl.edu), we have recently been granted the DFG-funded Clinical Research Unit “Male Germ Cells: from Genes to Function” (CRU326, http://male-germ-cells.de), and Joris Veltman (Newcastle University, Newcastle upon Tyne, UK and Radboud University Medical Centre, Nijmegen, The Netherlands) has very recently received the Wellcome Trust grant “Unravelling genetic causes and risk factors for severe male infertility”. The same investigators, together with Moira O’Bryan (Monash University, Melbourne, Australia) and Ewa Rajpert-De Meyts (Copenhagen University Hospital [Rigshospitalet], Copenhagen, Denmark), also recently founded the “International Male Infertility Genomics Consortium” (IMIGC, http://infertilegenome.org), which is aimed at the mutual exchange of clinical and genetic information to speed up the identification and interpretation of clinically relevant gene mutations. Such consortia have been established for several genetic diseases (such as intellectual disability) in the past and have without doubt demonstrated their benefits.

In summary, we are confident that major breakthroughs will be achieved in the near future concerning the genetic causes of male infertility. Initially, this will certainly cover azoospermia, but in the middle term will be broadened to multifactorial conditions such as oligozoospermia. First of all, this will greatly help to improve our understanding of the molecular biology of spermatogenic failure. Clinically most important, novel genetic diagnostic procedures, initially most likely comprehensive gene sequencing, will be introduced into diagnostic routine. This will allow for more precise risk estimates, better counselling of couples, and evidence-based treatment decisions. Ultimately, elucidating the causes underlying male infertility and corresponding phenotypes will pave the way for novel, personalised treatment regimens improving patient/couple care and offspring health.

## Practical conclusion

Screening for chromosomal aberrations and/or Y‑chromosomal azoospermia factor deletions are currently still at the forefront of genetic diagnostics for infertile men with disorders of spermatogenesis, i.e. oligo- or azoospermia. However, preliminary data from our screening study on three candidate genes have already shown that using specific gene analyses, the aetiological clarification of disturbed spermatogenesis can be significantly improved. Furthermore, clinically relevant results are expected from the studies currently underway, which could then be introduced into routine diagnostics within the framework of a gene panel analysis, thus improving the guidance and treatment given to men/couples.
